# Current landscape of drug induced retinal vasculitis

**DOI:** 10.1186/s12348-026-00619-5

**Published:** 2026-08-12

**Authors:** Sivaraman Bala Murugan, Aneena Anna Thomas, Rakshita Deepak Kene, Aditya Rajan, Anup Kelgaonkar, Vedhanayagi Rajesh, Anjana Somanath, Padmamalini Mahendradas, Gokulvasanth Deivasenathipathi, Parthopratim Dutta Majumder

**Affiliations:** 1 Department of Uveitis, Aravind Eye Hospital, Pondicherry, India; 2 Department of Uveitis and Ocular Immunology, Narayana Nethralaya, Bengaluru, India; 3 Department of Uvea & Intraocular Inflammations, Sankara Nethralaya, Chennai, India; 4 Uveitis service, Anant Bajaj Retina Institute, L V Prasad Eye Institute, Bhubaneswar, India; 5 Uvea Services, Aravind Eye Hospital, Madurai, India

## Abstract

Drug-induced retinal vasculitis (DIRV), a complex diagnostic entity, masquerades as infectious or primary autoimmune diseases. Delineating them is a daunting task. This update aims to consolidate clinical evidence focussing on characteristic “vascular signatures” using advanced multimodal imaging tools. Type III (immune complex-mediated) and Type IV (T-cell-mediated) hypersensitivity pathways were involved in diverse range of drugs causing DRIV. The critical therapeutic strategies are highlighted to prevent permanent ischemic damage. Advances in the recent metagenomic markers are added to aid the clinician from a practical standpoint to aid in preventive and personalised ophthalmic care.

## Introduction

Retinal vasculitis refers to inflammation of the retinal vascular wall and may occur in association with systemic inflammatory diseases, infections, malignancies, or adverse drug reactions. Drug-induced retinal vasculitis (DIRV) represents an uncommon but clinically significant subset, as failure to recognize a medication-related etiology may result in continued exposure to the offending agent, leading to progressive vascular damage and potentially irreversible visual loss. A wide range of pharmacologic agents have been implicated, including systemic antibiotics, antiepileptic medications, biologic therapies, immune checkpoint inhibitors, and locally administered intraocular drugs [1, 2]. Recent reports emphasize the growing incidence and clinical importance of DIRV associated with newer therapies, such as intravitreal Anti-Vascular Endothelial Growth Factor (Anti-VEGF) agents like brolucizumab [3, 4] and complement inhibitors such as pegcetacoplan [5]. 

The clinical manifestations of DIRV are diverse and frequently overlap with those of infectious retinitis, primary inflammatory retinal vasculitis, and retinal vascular occlusive disorders, often resulting in diagnostic uncertainty. Patients may present with retinal hemorrhages, vascular sheathing, capillary non-perfusion, retinal ischemia, and variable involvement of retinal arteries, veins, or both. Severe sight-threatening variants, such as vancomycin-associated hemorrhagic occlusive retinal vasculitis (HORV), can even masquerade as central retinal vein occlusions, further complicating the clinical picture [6]. The onset of disease is often delayed, occurring days to weeks after drug exposure, and may be more severe following re-exposure, supporting an immune-mediated pathogenesis rather than direct retinal toxicity.

### Method of literature search

This review addresses this lacuna by synthesizing “vascular signatures” and multimodal imaging hallmarks, moving beyond anecdotal evidence toward a standardized, clinical paradigm for the clinician.

To identify studies describing retinal vascular inflammation, the terms “drug-associated retinal vasculitis”, “drug-induced retinal vasculitis”, “medication-related retinal vasculitis”, “retinal vasculitis”, “occlusive retinal vasculitis”, “retinal vascular inflammation”, and “retinal vascular occlusion” were linked using the Boolean operator “OR”.

A second search set was constructed using names of drugs and therapeutic classes previously implicated in retinal vasculitis, including “brolucizumab”, “vancomycin”, “hemorrhagic occlusive retinal vasculitis”, “immune checkpoint inhibitors”, “nivolumab”, “pembrolizumab”, “ipilimumab”, “interferon”, “bisphosphonates”, “rifabutin”, “cidofovir”, “tumor necrosis factor inhibitors”, “etanercept”, “infliximab”, “adalimumab”, and “drug toxicity”, which were also combined using “OR”. The two search blocks were subsequently merged using the Boolean operator “AND” to identify relevant records. To maximize study retrieval, the bibliographies of eligible articles were manually reviewed for additional references. Publications written in English that reported drug-related retinal vasculitis in the form of case reports, case series, observational studies, or review articles were considered eligible for inclusion.

### Pathophysiology of drug-induced retinal vasculitis

The pathophysiologic mechanisms underlying DIRV are heterogeneous and incompletely understood, but most evidence supports immune-mediated vascular injury rather than direct endothelial toxicity [7]. Several immunologic mechanisms have been proposed, paralleling those described in systemic drug-induced vasculitis.

One proposed mechanism is a type III hypersensitivity reaction, in which circulating immune complexes formed between drug antigens and antibodies deposit within vascular walls, activate complement, and recruit inflammatory cells, resulting in endothelial damage and vascular occlusion. This mechanism is well described in systemic leukocytoclastic vasculitis and has been extrapolated to retinal vasculitis in selected drug reactions. The delayed onset following exposure and venular predominance seen in some cases support this hypothesis [8]. 

More recent evidence suggests that type IV (T-cell–mediated) hypersensitivity reactions may play a central role in certain drug-induced retinal vasculitides [9, 10]. In this model, drug antigens trigger cytotoxic T-cell activation, leading to endothelial injury through the release of pro-inflammatory cytokines, perforins, granzymes, and Fas–Fas ligand interactions [10, 11]. This mechanism is analogous to severe cutaneous adverse drug reactions such as Stevens–Johnson syndrome and toxic epidermal necrolysis and may explain delayed presentation, progression despite drug clearance, and more severe disease upon re-exposure [11, 12]. 

Endothelial injury from immune activation results in breakdown of the blood–retinal barrier, intravascular thrombosis, and widespread retinal ischemia. Secondary upregulation of vascular endothelial growth factor (VEGF) in response to ischemia contributes to neovascular complications, including retinal neovascularization and neovascular glaucoma. In advanced cases, ischemic damage may extend beyond the retina, involving the choroid and anterior segment structures [10, 13]. 

Importantly, the absence of systemic vasculitis does not exclude a drug-induced mechanism, as immune reactions may remain localized to the eye. The variability in clinical phenotype likely reflects differences in immune pathways activated by specific drugs, host susceptibility, and prior sensitization [7, 8]. 

### Antibiotics and anti virals

#### Vancomycin

Vancomycin is a glycopeptide antibiotic commonly reserved for the management of severe ocular infections caused by Gram-positive organisms. In ophthalmic practice, it is administered as fortified topical eye drops and through intravitreal or intracameral injections. Hemorrhagic occlusive retinal vasculitis (HORV) is a rare but severe postoperative inflammatory vasculopathy that has been most consistently linked to the use of intracameral vancomycin during intraocular surgery. HORV typically presents 1–21 days after injection following an initially uneventful postoperative course, often leading to delayed diagnosis. Visual acuity is frequently reduced at presentation, while early symptoms may be minimal. Despite treatment, visual outcomes are often poor, with many eyes progressing to severe visual impairment or no light perception [9, 14, 15]. 

The pathogenesis of HORV remains incompletely understood. Early theories proposed an immune complex–mediated type III hypersensitivity reaction to intracameral vancomycin, supported by the delayed onset of disease and the tendency for bilateral cases to exhibit earlier and more severe involvement of the second eye [9, 14]. However, histopathological studies of enucleated eyes failed to demonstrate immune complex deposition or leukocytoclastic vasculitis within the neurosensory retina. Instead, they revealed chronic non-granulomatous choroiditis with necrotic and hemorrhagic changes in the overlying retinal vasculature, accompanied by a predominantly CD4 + and CD8 + T-cell infiltrate with mast cells and eosinophils, while B cells and plasma cells were sparse [10]. These findings support a drug-induced, T-cell–mediated type IV hypersensitivity reaction, analogous to severe cutaneous adverse reactions such as Stevens–Johnson syndrome and toxic epidermal necrolysis associated with systemic vancomycin exposure [10–12]. Cytotoxic T-cell activation may lead to choroidal inflammation and secondary retinal necrosis, explaining the delayed presentation and absence of immune complexes. Consequently, some authors have proposed the term hemorrhagic occlusive choroidal and retinal vasculopathy (HOCRV), reflecting a primary choroidal process with secondary retinal involvement [10–12]. Additional findings, including anterior segment ischemia, iris and ciliary body necrosis, glomeruloid endothelial proliferation, and intravascular thrombosis, further support HORV as a localized drug-induced microangiopathy rather than a direct toxic effect [10, 13]. 

Clinically, HORV is characterized by widespread retinal hemorrhages, occlusive retinal vasculopathy predominantly affecting venules, and extensive retinal ischemia. Fluorescein angiography (FA) typically demonstrates large areas of capillary nonperfusion with staining or leakage at the margins of occluded vessels. Late optic disc hyperfluorescence may be present [9] Optical coherence tomography frequently reveals hyperreflectivity and thickening of the inner retinal layers consistent with ischemic injury, along with intraretinal cystic changes. As the disease progresses, ischemia-driven complications such as retinal neovascularization and neovascular glaucoma are common and contribute significantly to long-term visual morbidity [9, 14]. 

The differential diagnosis of HORV includes acute postoperative endophthalmitis, viral retinitis, toxic retinopathy, and central retinal vein occlusion. Considerable clinical overlap exists among these entities, making HORV a challenging diagnosis, particularly early in its course. Misdiagnosis is common, and systemic evaluation is often required to exclude infectious, inflammatory, or thrombotic causes of occlusive retinal disease [8, 9]. 

Despite prompt and aggressive therapy—including corticosteroids, panretinal photocoagulation, and intravitreal anti–vascular endothelial growth factor (anti-VEGF) agents—outcomes remain poor in many cases. Progressive ischemia may continue even with early intervention, and neovascular glaucoma may develop months to years after presentation [9, 14]. 

Given the severity and unpredictability of HORV, prevention remains paramount. Surgeons must carefully weigh the benefits of intracameral vancomycin for endophthalmitis prophylaxis against the potential risk of this devastating complication [10–13, 15, 16]. Re-exposure to vancomycin, particularly in the fellow eye or for suspected postoperative endophthalmitis, should be avoided due to the risk of immune sensitization and more severe disease [9, 14]. 

#### Gentamicin

Gentamicin is a potent broad-spectrum aminoglycoside antibiotic that is particularly effective against Gram-negative bacteria. However, owing to its narrow therapeutic index, even a small increase in intraocular concentration can result in significant retinal toxicity. McDonald et al. first described the effects of inadvertent intravitreal gentamicin injection in three patients in 1986 [17]. Long-term sequelae included rubeosis iridis, neovascular glaucoma, retinal pigmentary degeneration, and optic atrophy. Additional isolated reports have described multiple retinal vascular occlusions following subconjunctival gentamicin injection at the conclusion of 25-gauge vitrectomy [18]. These patients developed extensive perifoveal vascular disruption with areas of capillary non-perfusion on fluorescein angiography approximately 10 days after injection, accompanied by a decline in vision to hand movements.

It has been postulated that postoperative wound leak and hypotony may facilitate the migration of gentamicin from the subconjunctival space into the vitreous cavity, resulting in retinal toxicity. Given the potential for severe and irreversible retinal damage, extreme caution should be exercised when administering intraocular aminoglycosides, particularly gentamicin.

#### Amikacin

Amikacin is an aminoglycoside antibiotic commonly administered intravitreally for the treatment of endophthalmitis. Several case reports have described amikacin-associated macular ischemia following intravitreal injection in patients with postoperative endophthalmitis [19, 20]. In another report, a 26-year-old man with an intraocular foreign body received intravitreal amikacin in combination with vancomycin [21]. Postoperatively, his visual acuity was reduced to finger counting, and fundus examination revealed macular edema with loss of the foveal reflex. Fluorescein angiography confirmed macular ischemia, while optical coherence tomography showed increased retinal thickness with reduced intra retinal reflectivity.

The retinal toxicity of aminoglycosides is attributed to their preferential effects on the neurons and glial cells of the inner retina. Several factors may increase the risk of toxicity, including improper needle positioning during injection, particularly when the needle is directed toward the posterior pole or macula. Repeated injections may further increase the risk of retinal damage [22]. 

#### Cefuroxime

Cefuroxime is a second-generation cephalosporin antibiotic with broad-spectrum antimicrobial activity. In ophthalmic practice, cefuroxime is most commonly administered via intracameral and subconjunctival injection. Inadvertent administration of high-dose cefuroxime is a well-recognized cause of intraocular toxicity.

However, retinal toxicity has also been reported following administration of standard doses, suggesting that toxicity may not be exclusively dose dependent. There are also reports stating that it cannot be ruled out that even in normal dosing this drug can cause retinal toxicity. In a report by Zuo et al. where he presented 20 cases who had macular edema and serous detachment post usage of the drug intra operatively in cataract surgery [23]. But these patients have recovered with 20/20 vision in a week. Less common manifestations included retinal hemorrhages, retinal infarction, and optic nerve atrophy. In a study from Türkiye involving 245 eyes that received standard-dose intracameral cefuroxime following posterior capsular dehiscence, retinal vascular complications were reported [24]. Haemorrhagic retinal vasculitis noted in three of the patients who had received cefuroxime. These patients had drop in vision post operatively.

The exact mechanism remains unclear; however, cefuroxime has been proposed to induce overexpression of glutamate transporter proteins, potentially altering glutamate concentrations at outer plexiform layer synapses. Additional proposed mechanisms include disruption of the blood-retinal barrier, direct injury to photoreceptor and retinal pigment epithelial (RPE) cells, and dysfunction of the RPE sodium-potassium pump, resulting in retinal edema and toxicity. Reported risk factors include previous vitreoretinal surgery, zonular abnormalities, posterior capsular dehiscence, and secondary intraocular lens implantation, all of which may facilitate greater drug exposure to the posterior segment. Topical corticosteroids and non-steroidal anti-inflammatory drugs are commonly used and are often associated with favorable visual recovery.

#### Rifabutin

Rifabutin is a rifamycin derivative commonly used in the treatment of mycobacterial infections. Although rifabutin-associated hypopyon uveitis is well recognized, retinal vasculitis is uncommon. Ocular inflammation is more frequently reported at higher doses but may also occur at standard doses (300–450 mg/day), particularly in patients with low body weight, hepatic dysfunction, or concomitant use of CYP450 inhibitors such as clarithromycin or fluconazole [25, 26]. Symptoms typically develop weeks to months after initiation of therapy. Clinical manifestations include anterior uveitis with occasional retinal vasculitis, and fluorescein angiography may help determine the extent of posterior segment involvement [27, 28]. The pathogenesis remains uncertain but has been attributed to genetic susceptibility and hypersensitivity responses related to underlying mycobacterial infection [29]. Management consists of discontinuing rifabutin and initiating corticosteroid therapy. While anterior segment inflammation usually resolves rapidly, retinal vasculitis may follow a more prolonged clinical course [25, 29]. 

#### Cidofovir

Cidofovir is an antiviral agent used in the management of cytomegalovirus (CMV) retinitis. Posterior segment inflammation associated with cidofovir most commonly occurs in the setting of immune recovery uveitis (IRU) rather than direct drug toxicity. Cystoid macular edema develops in approximately 18–22% of eyes following immune recovery, typically when CD4 + counts exceed 50 cells/µL, and may occur between 3 and 48 weeks after treatment [30, 31]. Clinical findings include vitritis, perivascular infiltrates, and cystoid macular edema, particularly in eyes with extensive healed CMV lesions. The inflammatory response appears to be driven primarily by immune reconstitution directed against cidofovir-damaged ciliary epithelial antigens and antigens released from healed CMV lesions. This process is largely T-cell mediated and amplified by pro-inflammatory cytokines such as TNF-α, IFN-γ, and Interleukin(IL)-6 (IL-6), whereas direct drug toxicity appears to play a minor role [32]. Management includes discontinuation of cidofovir once sustained immune recovery has been achieved, together with periocular or systemic corticosteroids and topical non-steroidal anti-inflammatory drugs. Refractory cases may require additional immunosuppressive therapy such as mycophenolate mofetil or azathioprine. Continued antiretroviral therapy remains essential, and regular ophthalmic monitoring during immune recovery can substantially reduce the incidence of posterior segment complications [32, 33]. 

### Anti-VEGF

#### Brolucizumab

With the advent of Brolucizumab, it became popular with its superior drying effect and long interval dosing of 8–12 weeks. However, the adverse effects like retinal vasculitis and intraocular inflammations were unavoidable in some cases. Although, exact pathogenesis behind this phenomenon is not known, the existing literature reports vasculitis to be non-infectious, commonly observed in females, where the patients have received anti-VEGF injections in the past. Retinal vasculitis preceded by intraocular inflammation by few weeks. The HAWK and HARRIER trials reported Intraocular Inflammation (IOI) developing in 7 eyes with retinal vasculitis and vascular occlusion 3–49 days post injection, without any gender predilection [34]. 

Brolucizumab differs from other anti- VEGF by being the smallest molecule with no Fc portion. Importantly, it is a drug with preexisting anti- drug antibodies (ADA) unlike other anti-VEGF molecules. Small size of Brolucizumab allows its higher molar concentration which leads to local immune reaction and inflammation. Type III hypersensitivity reaction or Arthus reaction is thought to occur with monoclonal antibodies including anti VEGF therapies [35]. Similarly, the high concentration of the drug and formation of ADA in the circulation can lead to type III hypersensitivity reaction in such cases. Additionally, brolucizumab is associated with higher quantities of pretreatment ADA compared to ranibizumab and aflibercept.

In a review article by Wykoff et al. [36], 70 eyes of 63 patients with retinal vasculitis or retinal occlusion post Brolucizumab injection were included. The mean age of patients developing this adverse event was 77.6 years and 77.8% of them were women. Approximately 46% of them had received one injection of Brolucizumab and rest all received two to three injections. Half of the cases were associated with deterioration of vision. Young patients with non-occlusive retinal vasculitis had no vision loss.

Witkin et al. [37] analysed 26 eyes of 25 patients with retinal vasculitis and they reported retinal vascular occlusion and retinal ischemia on examination or imaging in 83–88% cases, highlighting that most events are occlusive. The common features on examination were predominantly arterial involvement, although combined involvement was noted in 67% cases. Fundus features were arterial occlusion/ sheathing/ boxcarring, venous dilatation or beading, retinal whitening and sparse retinal hemorrhages.

In an isolated case report by Onagawa et al. [38], vasculitis was reported post two injections of brolucizumab in a patient on treatment for polypoidal choroidal vasculopathy. Patient was treated with sub tenons triamcinolone acetonide injection, topical and oral steroids for almost 4 months. However, patient had recurrence of anterior chamber inflammation with scleritis after the resolution of retinal vasculitis, which was treated with topical steroids.

Iyer et al. [39] reported a case of 76-year-old woman, who was switched to treatment with Brolucizumab due to non-response to other anti VEGFs for neovascular Age-Related Macular Degeneration (AMD). After the third loading dose patient had intense inflammation presenting as anterior chamber reaction 0.5+, vitreous debris, arterial plaques, mild vascular sheathing and boxcarring temporally. Her symptoms subsided with topical prednisolone acetate. However, she developed recurrence of inflammation three weeks post treatment with ranibizumab. Examination in right eye revealed fine keratic precipitates, anterior chamber cells 2 + significant vitreous haze and debris with occlusive vasculitis. Treatment was initiated with high dose oral pulse methyl prednisolone and topical difluprednate. However, due to persistence of symptoms after 3 days of therapy, patient underwent pars plana vitrectomy with intravitreal injection triamcinolone. The vitreous biopsy showed no evidence of infectious agent, but chronic inflammatory infiltrate.

Kotoaka et al. described three Japanese patients with neovascular age-related macular degeneration who developed intraocular inflammation and retinal vasculitis 11–18 days after a first intravitreal brolucizumab injection, including one treatment‑naïve and two switch cases from aflibercept. All eyes showed anterior chamber cells, keratic precipitates, vitreous haze, peripheral retinal hemorrhages, and ultra-widefield fluorescein angiographic evidence of segmental vascular leakage, with vascular sheathing and peripheral nonperfusion in two cases. Systemic oral prednisolone combined with subtenon triamcinolone and topical betamethasone led to rapid improvement in vascular leakage and recovery of visual acuity in all three eyes within six weeks, although some vascular filling defects persisted.

#### Faricimab

Faricimab is a bispecific monoclonal antibody targeting both vascular endothelial growth factor-A (VEGF-A) and angiopoietin-2 (Ang-2). Although faricimab-associated retinal vasculitis is rare, with an estimated incidence of 0.17 cases of occlusive retinal vasculitis per 10,000 injections, it remains an important vision-threatening complication. This incidence is substantially lower than that reported with brolucizumab but higher than that observed with conventional anti-VEGF agents such as aflibercept [40, 41]. Patients typically present with acute painless visual loss and floaters days to weeks after injection. Clinical examination may reveal anterior chamber cells, keratic precipitates, vitritis, retinal hemorrhages, and vascular sheathing. Fluorescein angiography demonstrates extensive vascular leakage in non-occlusive cases, whereas occlusive retinal vasculitis is characterized by capillary nonperfusion and may result in irreversible visual loss [40, 42]. The pathogenesis remains incompletely understood but appears to involve immune sensitization, with mild intraocular inflammation progressing to severe occlusive vasculitis upon re-exposure, suggesting a T-cell-mediated mechanism. Alternative hypotheses include anti-drug antibody formation against the bispecific molecule, leading to immune complex formation, complement activation, and vascular endothelial injury [41, 42]. Management involves immediate cessation of faricimab and treatment with systemic corticosteroids, often preceded by pulse intravenous methylprednisolone in severe cases. While non-occlusive vasculitis generally responds well to corticosteroid therapy, occlusive disease carries a guarded visual prognosis. Following resolution, switching to a low-immunogenicity anti-VEGF agent such as aflibercept is recommended [40, 42–44]. 

### Immunomodulators

#### Pegcetacoplan

Pegcetacoplan is a polyethylene glycolated (PEGylated) peptide which can block complement factor C3. Complement C3 plays a key role in the pathogenesis of both exudative and advanced atrophic age-related macular degeneration (AMD). The United States Food and Drug Administration has approved for intravitreal use of this drug in geographic atrophy in AMD. Within two months of approval, the American Society of Retina Specialists (ASRS) Research and Safety in Therapeutics Committee began receiving reports of severe post-injection ocular inflammation [45]. 

A total of 32 eyes from 28 patients developed intraocular inflammation following intravitreal pegcetacoplan injection. Among these, 17 eyes of 15 patients fulfilled the criteria for pegcetacoplan-associated retinal vasculopathy. Clinically these cases were analysed with fundus photography, Fluorescein angiography and OCT. The severity of vasculopathy ranged from mild inflammation to severe occlusive disease. Venular involvement was observed in 11 eyes, of which 8 also demonstrated arteriolar involvement, occlusive retinal vasculopathy, and peripheral ischemia. Macular ischaemia was noted in five of these eyes. Management included topical, systemic, and intravitreal corticosteroids. Six eyes had evidence of anterior segment neo vascularization and 2 eyes needed enucleation.

The reason postulated for such inflammation following injection in these patients is that, although PEG chains improve the molecule’s pharmacokinetic properties and efficacy, antibodies to these PEG chains formed due to previous exposure, can lead to hypersensitivity reaction, resulting in intraocular inflammation and retinal vasculitis. It is also postulated that the intravitreal injection of the drug dysregulates the complement system, which may lead to immune complex aggregation and vascular damage to retina in these patients.

Another report described two patients who developed severe inflammation and profound vision loss nine days after their first pegcetacoplan injection, both of whom subsequently underwent enucleation for pain control [46]. Histologic examination of the enucleated eyes revealed extensive vascular thrombosis, retinal hemorrhages and necrosis, and a dense inflammatory infiltrate in the uvea and there was notable absence of inflammatory infiltrate from the retina. Based on these findings, the authors proposed the term drug-induced immune-mediated retinal vasculopathy and choroiditis (DIRVAC) to describe this rare complication of pegcetacoplan therapy.

#### Immune checkpoint inhibitors

Immune checkpoint inhibitors (ICIs) are monoclonal antibodies used in cancer immunotherapy that block inhibitory proteins on T cells, such as Cytotoxic T-Lymphocyte-Associated Protein 4 (CTLA-4), Programmed Cell Death Protein 1 (PD-1), or Programmed Death-Ligand 1 (PD-L1), to prevent immune suppression and enhance the body’s antitumor response [47]. These proteins act as “brakes” on the immune system, signaling T cells to halt activation when binding partner proteins on antigen-presenting cells or tumor cells; by disrupting this interaction, ICIs unleash cytotoxic T cells to target and destroy cancer cells more effectively. ICIs such as anti-CTLA-4 (e.g., ipilimumab) and anti-PD-1/PD-L1 agents (e.g., pembrolizumab, durvalumab), can induce retinal vasculitis as a rare but vision-threatening immune-related adverse event by disrupting immune tolerance mechanisms [48]. These drugs block inhibitory receptors on T cells, unleashing hyperactive cytotoxic responses against tumor antigens that inadvertently cross-react with ocular vascular endothelium (Type 1 mechanism) or trigger bystander inflammation in predisposed eyes, leading to perivascular sheathing, capillary leakage, cystoid macular edema, and potential occlusive vasculopathy [49–51]. This T-cell mediated endothelial damage mimics endogenous uveitides, often confirmed by fluorescein angiography showing diffuse leakage, and responds to corticosteroids while allowing ICI continuation in select cases [49–51]. 

Rojas-Pineda et al. [52] reported bilateral retinal vasculitis in a 60-year-old man with esophageal squamous cell carcinoma treated with tislelizumab, an anti-PD-1 inhibitor, manifesting initially as asymptomatic peripheral intraretinal haemorrhages confirmed by fluorescein angiography. Corticosteroid therapy achieved control without therapy interruption, yet relapse with subfoveal fluid and vision loss ensued four months post-taper, with partial resolution upon reinitiation but incomplete visual recovery.

Kim et al. [50] described bilateral panuveitis and retinal vasculitis in a 71-year-old man with metastatic squamous cell lung carcinoma two weeks after initiating pembrolizumab, featuring arterial and venous involvement managed by temporary discontinuation, oral corticosteroids, and sub-Tenon triamcinolone, allowing therapy restart upon subsidence. Andrade et al. [51] reported retinal vasculitis in a 64-year-old man with non-small cell lung cancer after 20 months of durvalumab, presenting with unilateral vision loss, papillitis, cystoid macular edema, and phlebitis on wide-field fluorescein angiography, which resolved fully with intravenous methylprednisolone boluses and taper without halting immunotherapy.

In another case report, Tsui and Gonzales [49] detailed retinal vasculitis in a 49-year-old man with chronic myeloid leukemia five months post-ipilimumab initiation following anterior uveitis, showing left eye cystoid macular edema and capillary leakage responsive to topical steroids, oral prednisone, and intravitreal dexamethasone implant.

#### TNF-α inhibitors

Tumor necrosis factor-alpha (TNF-α) inhibitors, including infliximab, adalimumab, and etanercept, are widely used in the treatment of inflammatory and autoimmune disorders but have paradoxically been associated with retinal vasculitis. Ocular adverse events occur in approximately 3.5–12% of patients receiving anti-TNF therapy, although retinal vasculitis represents a rare manifestation and appears to occur more commonly in women [53]. The latency period is highly variable, ranging from several months to years, and onset often coincides with remission of the underlying systemic disease [54]. Clinical manifestations include anterior uveitis, vitritis, retinal vasculitis, retinal ischemia, optic neuritis, and, in severe cases, neovascular complications leading to vitreous hemorrhage [55]. Several mechanisms have been proposed, including a dysregulated *T helper type 1/2 (*Th1/Th2) cytokine balance with increased type I interferon production, formation of anti-drug antibody immune complexes resulting in type III hypersensitivity reactions, and loss of TNF-α-mediated regulatory T-cell differentiation and immune tolerance [56–59]. Management requires discontinuation of the offending agent together with systemic and local corticosteroid therapy. Retinal neovascularization may require laser photocoagulation, and recurrence following cessation of TNF-α inhibitors is uncommon [54]. 

### Miscellaneous DIRV

Several other pharmaceutical agents have been reported to be associated with DIRV. Propylthiouracil has been associated with anti-neutrophil cytoplasmic antibodies (ANCA)-mediated retinal vasculitis through loss of immune tolerance, facilitated by myeloperoxidase (MPO)-binding metabolites and neutrophil extracellular traps (NETs), an important component of innate immunity [60]. Gao et al. [61] elucidated that higher MPO-ANCA titers correlate with the severity and occlusive nature of this pauci-immune vasculitic process. Bone marrow abnormalities and hemophagocytosisis established by the recent larger series [62].

Estrogen-based therapies induce a pro-thrombotic retinal vasculopathy. Estrogen elevates clotting factors and decreases Antithrombin III activity, thereby predisposing patients to thrombotic vascular events. While idiopathic in appearance, the temporal link to hormonal therapy is a documented trigger for these inflammatory vascular events. Management requires immediate discontinuation of the hormone and ruling out underlying thrombophilic mutations to prevent contralateral involvement. (Table 1) [63, 64].

Phenytoin-induced retinal vasculitis can occur within the settings of the DRESS syndrome (a T-cell mediated Type IV systemic hypersensitivity response) or, alternatively, manifest as a drug-induced ANCA-associated vasculitis (DI-AAV) driven by a hapten-mediated Type III hypersensitivity pathway, as summarized in Table 3. The characteristic ocular findings include bilateral retinal periphlebitis and retinal microinfarcts. Management necessitates immediate drug cessation and systemic corticosteroids to address the underlying multi-organ inflammatory cascade, as ocular signs often herald severe systemic decompensation.

Talc retinopathy results from intravenous injection of crushed oral tablets containing magnesium silicate filler particles. Clinically, it presents with bilateral, glistening yellow-white end-arteriolar particles. Chronic occlusion induces peripheral ischemia and secondary neovascularization, mimicking proliferative sickle cell retinopathy. Diagnosis is confirmed via fluorescein angiography showing peripheral non-perfusion. Prognosis depends on drug cessation; however, existing crystals remain permanent, often requiring laser photocoagulation for complications.(Table 2) [65–67].

Bisphosphonate-induced retinal vasculitis is thought to result from a robust inflammatory response triggered by inhibition of farnesyl pyrophosphate synthase within the mevalonate pathway, leading to T-cell activation. Subsequent release of pro-inflammatory cytokines, including interleukin-6 (IL-6) and tumor necrosis factor-alpha (TNF-α), may disrupt the blood-retinal barrier, resulting in perivascular inflammation and capillary leakage. Clinically, it manifests within 72 h of the first dose and presents as diffuse periphlebitis and leakage. Management requires immediate cessation and aggressive topical or systemic corticosteroids to prevent permanent macular edema. Interestingly, the nitrogen-containing bisphosphonates (Zoledronate/Pamidronate) have a higher tendency to cause DIRV than non-nitrogen agents (Etidronate). Optical coherence tomography angiography (OCT-A) may demonstrate vessel dilation and vascular engorgement, in contrast to the flow voids observed in talc retinopathy and phenytoin-associated vasculitis.

Rituximab-associated retinal vasculitis has been reported as a paradoxical inflammatory reaction occurring approximately 7–14 days after infusion. Pathogenesis involves anti-rituximab immune complexes sequestering within precapillary arterioles, triggering focal injury. In addition to perivascular sheathing, OCT-A uniquely captures peripheral “flow voids” stemming from immune-mediated stasis. This unique ischemic signature differs from the vessel dilation seen in bisphosphonate-related cases. Prompt drug withdrawal and aggressive corticosteroids remain the definitive management.

Hydralazine-induced DIRV manifests as a localized retinal vasculitis, due to aberrant induction of ANCA. This autoimmune trigger leads to small-vessel vasculitis, that manifests clinically as diffuse perivascular sheathing and intraretinal hemorrhages. Paradoxically, symptoms emerge only after prolonged exposure, reflecting a chronic, smoldering inflamed state. Diagnosis requires correlating positive MPO-ANCA titers with segmental leakage with multimodal imaging tests.While drug cessation is mandatory, recalcitrant presentations often demand systemic immunosuppression beyond steroids to achieve quiescence.

Methamphetamine-associated DIRV presents as an occlusive vasculitis, precipitated by the systemic sympathetic surge and secondary vasospasm [68]. Pathophysiologically, the drug causes profound endothelial dysfunction and localized ischemia as evidenced by cotton wool spots, arteriolar narrowing, and intraretinal hemorrhages. Acute hypertensive crises and direct vascular toxicity may contribute to a necrotizing vasculitic process [68]. Diagnosis depends on identifying characteristic clinical profile and capillary non-perfusion on Fluorescein Angiography. Management needs immediate drug cessation, though visual recovery remains guarded due to permanent inner retinal infarction [68].

Cocaine-associated retinal vasculitis most commonly manifests as an acute occlusive vasculopathy secondary to intense adrenergic stimulation and vasospasm [68]. This results in branch or central retinal artery occlusions as a manifestation of localized vasculitis. With embolic events as a differential diagnosis, cocaine specifically drives a necrotizing vascular damage through both acute hypertensive crises and direct toxic effects on the microvasculature [68]. Diagnosis is established by fluorescein angiography, which typically reveals segmental capillary non-perfusion and arterial narrowing. Management strategies includes systemic stabilization and drug cessation, but the prognosis for visual restoration remains poor due to infarction of retina [68].

Mitomycin-C (MMC), though typically restricted to ocular surface surgery, can inadvertently induce severe iatrogenic retinal vasculitis through intraocular penetration or systemic absorption. It characteristically triggers a delayed-type hypersensitivity or direct cytotoxic insult on the tunica adventitia, leading to extensive periphlebitis and occlusive vasculopathy. The clinical hallmarks are evident as profound vascular sheathing and cotton wool spots, that progress to widespread capillary non-perfusion proven by fluorescein angiogram. As a salient feature MMC-induced damage is distinctly progressive and refractory unlike the transient DIRV of bisphosphonates. Management paradigms includes aggressive corticosteroid therapy, but the visual prognosis is guarded if significant macular ischemia or neovascular glaucoma are noted.

### Work-up of a patient with DIRV

The evaluation of DIRV begins with a thorough systemic and medication history, as the diagnosis is largely one of exclusion. Particular attention should be paid to recent exposure to drugs known to cause ocular inflammation. Since the clinical presentation may closely resemble infectious or autoimmune retinal vasculitis, alternative etiologies must be carefully excluded before attributing the findings to a drug-induced process.

A comprehensive ophthalmic examination should be performed to identify the pattern and extent of inflammation. Common clinical findings include vascular sheathing, retinal hemorrhages, cotton-wool spots, and retinal ischemia manifesting as retinal whitening. Distinguishing between occlusive and non-occlusive vasculitis is important, as certain drugs demonstrate characteristic vascular phenotypes. For example, vancomycin is associated with HORV, whereas pegcetacoplan-associated vasculopathy may occasionally be accompanied by choroiditis [69]. Recognition of such associated findings may provide important clues to the underlying offending agent.

Multimodal retinal imaging plays a pivotal role in diagnosis and monitoring. FA remains the gold standard for evaluating retinal vasculitis, demonstrating vessel wall staining, vascular leakage, and, in occlusive forms, areas of capillary non-perfusion. Ultra-widefield FA is particularly useful for detecting peripheral vascular involvement that may otherwise be missed on conventional imaging. OCT and OCTA provide complementary information regarding retinal structure and perfusion [70]. Common findings include retinal thickening, cystoid macular edema, and inner retinal ischemic changes, while OCTA allows non-invasive assessment of capillary density and macular perfusion.

Systemic and laboratory investigations are essential to exclude inflammatory and infectious causes of retinal vasculitis. Initial evaluation should include inflammatory markers such as erythrocyte sedimentation rate (ESR) and C-reactive protein (CRP), along with autoimmune screening tests including antinuclear antibodies (ANA) and antineutrophil cytoplasmic antibodies (ANCA). Additional investigations should be guided by clinical suspicion and may include serum angiotensin-converting enzyme (ACE) levels for sarcoidosis and other disease-specific tests.

Exclusion of infectious etiologies is particularly important, as management differs substantially from that of non-infectious vasculitis. Tuberculosis, syphilis, and viral retinitis represent key differential diagnoses. Tuberculosis screening may include a Mantoux test and/or interferon-gamma release assays such as QuantiFERON-TB Gold. Syphilis should be evaluated using both treponemal and non-treponemal tests according to the reverse screening algorithm. In patients with suspected infectious endophthalmitis, vitreous biopsy is often warranted, as bacterial pathogens such as Streptococcus and Staphylococcus species, as well as fungal organisms including Candida species, may present with retinal vasculitis as part of the disease spectrum. In cases where viral retinitis is suspected, vitreous samples should be analyzed using polymerase chain reaction (PCR) testing for herpes simplex virus (HSV), varicella-zoster virus (VZV), and cytomegalovirus (CMV).

### Management

Management of DIRV begins with prompt discontinuation of the offending drug [71]. Following exclusion of infectious etiologies, patients with severe inflammation are typically treated with high-dose systemic corticosteroids, while local corticosteroid injections may be useful in selected cases. Intravitreal Anti-VEGF treatment has to be used in cases with macular edema. Pars plan vitrectomy is usually done in cases where an early lavage can help removing the offending drug when it is secondary to local treatment or in patients where it will help to clear up the vitreous haemorrhage in late stages. Pan retinal photo coagulation may be needed in occlusive vasculitis cases. Long standing cases may need treatment with immunosuppressive and in rare cases even newer biologics. Management must be tailored depending on the offending drug and the type and degree of inflammation the patient presents to us.

Emerging evidence suggests that host genetic susceptibility may influence the development of DIRV, although current data are insufficient to establish a direct causal relationship. Brolucizumab-associated retinal vasculitis illustrates this concept, as up to 43.7% of treatment-naïve patients possess baseline anti-drug antibodies, yet only a small proportion develop retinal vasculitis [72] This discrepancy is explained by a transition from humoral recognition to a cellular Type IV delayed-type hypersensitivity (DTH) response. The Cholesteryl Ester Transfer Protein (CETP) gene (rs3764261) is a confirmed pharmacogenomic marker: the T-allele confers protection, whereas C-allele carriers are at risk [73]. The marked female predominance reported in brolucizumab-associated vasculitis further suggests a possible sex-related immunological predisposition. In contrast, vancomycin-associated hemorrhagic occlusive retinal vasculitis appears to result primarily from delayed immune sensitization to vancomycin-protein complexes, although definitive genetic associations have yet to be identified.

Proteomic studies have provided additional insights into the immunopathogenesis of DIRV. Analysis of vitreous samples may help differentiate inflammatory vasculitis from infectious endophthalmitis while identifying potential molecular pathways involved in disease development. Current evidence highlights the importance of the IL-23/IL-17 axis, in which IL-23 promotes differentiation of pathogenic Th17 lymphocytes that subsequently produce IL-17 A. This cytokine disrupts vascular integrity through downregulation of tight-junction proteins such as claudin-5 and occludin, resulting in increased vascular permeability, leukocyte extravasation, and breakdown of the blood-retinal barrier [9]. Elevated levels of CXCL8 (IL-8) further promote recruitment of neutrophils and monocytes, contributing to the acute inflammatory response. In brolucizumab-associated intraocular inflammation, proteomic studies also support a type III hypersensitivity mechanism involving anti-drug antibody-mediated immune complex deposition and activation of the classical complement pathway, reflected by elevated vitreous concentrations of C1q, C3, and C5a.

These observations may have future therapeutic implications. Screening for genetic susceptibility markers such as CETP polymorphisms and anti-drug antibody profiles could potentially identify individuals at increased risk for DIRV. Similarly, the demonstration of complement activation raises the possibility of targeted therapies directed against C3 or C5 in severe occlusive disease. Future studies may also evaluate the role of cytokine-specific therapies targeting IL-23 or IL-17, such as guselkumab or secukinumab, particularly in patients with corticosteroid-refractory disease.

The practical clinical implications of drug-induced retinal vasculitis and the characteristic vascular signatures associated with individual agents are summarized in Tables 1, 2 and 3. This review has several limitations. Owing to the rarity of DIRV, the available evidence is derived predominantly from case reports and small case series, introducing significant publication and reporting bias, as severe or unusual presentations are more likely to be reported. Consequently, the true incidence, dose-response relationships, and timelines of immune sensitization remain incompletely defined. In addition, the absence of standardized multimodal imaging in many earlier reports introduces detection bias and may have resulted in under-recognition of milder or subclinical forms of retinal vasculitis.

## Conclusion

DIRV remains a diagnostic frontier where clinical vigilance must match therapeutic innovation. Early recognition through multimodal imaging is essential to halt irreversible ischemia and preserve visual function. Future research should prioritize the establishment of prospective registries to mitigate reporting bias and the correlation of quantitative OCT-Angiography metrics with drug-serum levels. Identifying genetic predispositions for these idiosyncratic reactions will ultimately transform our reactive management into a proactive, standardized paradigm for ocular safety.

**Table 1:** Summary of DIRV

Drug / Agent	Ophthalmic Signature & MMI Findings	Salient Treatment Protocol	Naranjo Score (Causality)	Revised Naranjo Score (Causality)	Risk of Bias (RoB)
Vancomycin (Intracameral)	HORV: Delayed-onset occlusive vasculitis. FFA: Peripheral arteriolar “pruning.”	Stop drug. IVT/Systemic Steroids + Early PPV + Anti-VEGF for NVE.	9 (Definite)	7 (Probable)	Moderate (Large series, but retrospective)
Brolucizumab	IOI with Vasculitis: Focal arterial staining. OCT: Hyper-reflective foci.	Intense Steroids (Topical/Sub-Tenon/Systemic). Hold future doses.	9 (Definite)	7 (Probable)	Low (Phase III/IV Clinical Trial data)
Aminoglycosides (Gentamicin)	Direct Toxicity: Macular whitening, cherry-red spot. ERG: Selective b-wave loss.	Urgent PPV to washout drug. Prognosis usually poor ($<$20/400).	9 (Definite)	6 (Probable)	High (Mostly case reports of errors)
Phenytoin	ANCA+ Vasculitis: Peripheral sheathing. Lab: p-ANCA positive (Anti-MPO).	Stop Phenytoin. Switch to alternative (e.g., Levetiracetam) + Systemic Steroids.	7 (Probable)	7 (Probable)	High (Rare systemic-ocular reports)
Propylthiouracil (PTU)	ANCA+ Vasculitis: Bilateral leakage. Lab: High p-ANCA titers.	Switch to Methimazole. Systemic Corticosteroids/Cyclophosphamide.	8 (Probable)	8 (Probable)	High (Rare case reports)
Rifabutin	Pseudohypopyon: Dense AC cells. FFA: Diffuse perivenous leakage.	Stop Rifabutin. Topical Steroids + Cycloplegics. Excellent recovery.	9 (Definite)	7 (Probable)	Low (Dose-dependent clinical hallmark)
Cidofovir	Uveitis-Hypotony: Ciliary body shutdown. UBM: Ciliary body thinning.	Probenecid (prophylaxis). Topical Steroids + NSAIDs.	8 (Probable)	8 (Probable)	Moderate (Strong longitudinal cohort data)
Immune Checkpoint Inhibitors	VKH-like: Subretinal fluid. ICGA: Multiple hypofluorescent spots.	Periocular/Systemic Steroids. Temporary drug holiday if severe.	7 (Probable)	7 (Probable)	Moderate (Emerging registries)
Pegcetacoplan	Occlusive Vasculitis: Rapid onset post-IVT. FFA: Vascular wall staining.	Intense Steroids. Monitoring for secondary neovascularization.	6 (Probable)	6 (Probable)	High (Post-marketing surveillance)
Talc (IV use)	Embolic: Intraluminal glistening crystals. OCTA: Micro-flow voids.	Addiction Counseling. PRP for secondary neovascularization.	9 (Definite)	6 (Probable)	Moderate (Diagnostic imaging is pathognomonic)

AC: Anterior Chamber, ANCA: Antineutrophil Cytoplasmic Antibodies, ERG: Electroretinography, FFA: Fundus Fluorescein Angiography, HORV: Hemorrhagic Occlusive Retinal Vasculitis, ICGA: Indocyanine Green Angiography, IOI: Intraocular Inflammation, IVT: Intravitreal Therapy, MMI: Multimodal Imaging, MPO: Myeloperoxidase, NVE: Neovascularization Elsewhere, OCT: Optical Coherence Tomography, OCTA: Optical Coherence Tomography Angiography, PPV: Pars Plana Vitrectomy, PRP: Panretinal Photocoagulation, PTU: Propylthiouracil, UBM: Ultrasound Biomicroscopy, VEGF: Vascular Endothelial Growth Factor, VKH: Vogt-Koyanagi-Harada disease

**Table 2:** Clinical pointers for DIRV

Clinical pointer	Possible DIRV	Treatment
“Fire and Ice” (Hemorrhage + Ischemia)	Vancomycin (HORV)	Stop drug; IVT Steroids; PPV.
Glistening perimacular spots	Talc / IV Drug Use	OCTA; Systemic workup for emboli.
Bilateral sheathing + Renal issues	PTU / Phenytoin	Order p-ANCA; Switch meds.
Hypopyon + Diffuse Venous Leakage	Rifabutin	Stop drug; Topical Steroids.

ANCA: Antineutrophil Cytoplasmic Antibodies, HORV: Hemorrhagic Occlusive Retinal Vasculitis, IVT: Intravitreal Therapy, OCTA: Optical Coherence Tomography Angiography, PPV: Pars Plana Vitrectomy, PTU: Propylthiouracil

**Table 3:** Clinico-pathological correlations of DIRV

Feature	Gentamicin (Toxicity)	PTU / Phenytoin (DI-AAV)	Talc (Embolic)	Hormones (OCP/HRT)	Bisphosphonates
1. Hypothesis	Direct mitochondrial/oxidative toxicity to inner retina.	Hapten-mediated p-ANCA (Type III) hypersensitivity.	Mechanical precapillary arteriolar occlusion.	Hypercoagulable state (Factors VII/X/Fibrinogen).	Cytokine-mediated (Type IV) acute inflammation.
2. Vision Range	NLP to 20/400 (Usually catastrophic).	Variable; depends on ischemic severity.	20/20 to 20/200 (Peripheral focus).	20/50 to HM (if CRVO occurs).	20/40 to 20/400 (Steroid responsive).
3. Demographics	Post-op (Endophthalmitis/Trauma).	Long-term use; elderly or hyperthyroid pts.	IV drug users (crushed oral tablets).	Females; smokers; age 20–50.	Post-infusion (Osteoporosis/Oncology).
4. Vessels Involved	Global arterial/capillary infarction.	Small vessel vasculitis (Capillaries).	Precapillary Arterioles.	Central or Branch Retinal Veins.	Diffuse retinal vasculature (sheathing).
5. Macular Involvement	Cherry-red spot; profound edema/thinning.	Ischemic maculopathy (if systemic).	Late-stage macular edema.	Cystoid Macular Edema (CME).	CME and perivascular leakage.
6. Clinical Features	Massive whitening; “Silent” ERG (B-wave).	Sheathing; hemorrhages; p-ANCA (+).	Glistening end-arteriolar crystals.	Venous tortuosity; flame hemorrhages.	Acute uveitis; scleritis; sheathing.
7. OCT/OCTA	Inner retinal hyper-reflectivity $\rightarrow$ Thinning.	Segmental capillary dropout.	Superficial crystalline reflectors.	Increased CFT; venous engorgement.	Diffuse leakage; perivascular cysts.
8. Risk Factors	Overdose; clear-cornea leakage; surgery.	Long duration of treatment ($>1$ year).	History of IVDU; pulmonary shunts.	Smoking; Factor V Leiden; dehydration.	IV administration (Zoledronate).
9. Prognosis	Poor; permanent inner retinal necrosis.	Fair if drug stopped + steroids.	Guarded; risks neovascularization.	Good to Fair with cessation.	Good if recognized early.
10. Neovascular.	Rare (Total atrophy).	Possible peripheral NV.	High (Sea-fan NV).	Moderate (post-ischemic CRVO).	Rare.
11. Treatment	Observation; Glaucoma management.	Stop drug; High-dose steroids/Rituximab.	Stop drug; Peripheral Laser (PRP).	Stop hormones; Anticoagulation.	Stop drug; Topical/Systemic steroids.
12. Level of Evidence	High (Ophth. Literature).	Moderate (Ophth. Case Series).	High (Clinicopathologic).	Moderate (Ophth. Cohort).	Moderate (Ophth. Database).

DI-AAV: Drug-Induced ANCA-Associated Vasculitis, CFT: Central Foveal Thickness, CME: Cystoid Macular Edema, CRVO: Central Retinal Vein Occlusion, ERG: Electroretinography, HM: Hand Movements, HRT: Hormone Replacement Therapy, IVDU: Intravenous Drug User, NLP: No Light Perception, NV: Neovascularization, OCP: Oral Contraceptive Pill, OCT: Optical Coherence Tomography, OCTA: Optical Coherence Tomography Angiography, PRP: Panretinal Photocoagulation, PTU: Propylthiouracil

## Data Availability

This manuscript does not report data generation. All literature analysed is available through public databases cited in the references.
